# Photodynamic dye adsorption and release performance of natural zeolite

**DOI:** 10.1038/srep45503

**Published:** 2017-03-31

**Authors:** Vladimir Hovhannisyan, Chen-Yuan Dong, Shean-Jen Chen

**Affiliations:** 1 College of Photonics, National Chiao Tung University, Tainan 711, Taiwan; 2 Department of Physics, National Taiwan University, Taipei 10617, Taiwan; 3 Advanced Optoelectronic Technology Center, National Cheng Kung University, Tainan 701, Taiwan

## Abstract

Clinoptilolite type of zeolite (CZ) is a promising material for biomedicine and pharmaceutics due to its non-toxicity, thermal stability, expanded surface area, and exceptional ability to adsorb various atoms and organic molecules into micropores. Using multiphoton microscopy, we demonstrated that individual CZ particles produce two-photon excited luminescence and second harmonic generation signal at femtosecond laser excitation, and adsorb photo-dynamically active dyes such as hypericin and methylene blue. Furthermore, the release of hypericin from CZ pores in the presence of biomolecules is shown, and CZ can be considered as an effective material for drug delivery and controlled release in biological systems. The results may open new perspectives in application of CZ in biomedical imaging, and introducing of the optical approaches into the clinical environment.

One of the most important areas of medical sciences and pharmaceutics is the incorporation of drugs into multifunctional carriers for controlled delivery of bioactive agents^1^. Targeted drug transport and following release with the controlled rate should reduce adverse effects and treatment period. Over the past few decades, biomedical and material sciences developed new nanostructured porous materials and new approaches in controlled drug delivery, optimization of treatment process, and enhancement of efficiency of health-care products^2^,^3^,^4^,^5^,^6^,^7^. Among the promising materials for drug delivery, as well as for biomedical sensing and imaging are zeolites.

Natural zeolites (ZLs) are widespread low-cost porous minerals, which unique surface and structural properties have been exploited in industrial^8^,^9^,^10^, agricultural^10^,^11^, environmental^12^,^13^,^14^,^15^,^16^, and biomedical technologies^17^. Due to exciting adsorption, cation-exchange, dehydration–rehydration, and catalytic shape selectivity, ZLs are used as filler in paper, in the take-up of radioactive Cs and Sr from nuclear waste and fallout, as soil amendments in agronomy and horticulture, in the removal of ammonia from drinking water and municipal wastewater, as filters in kidney-dialysis units, as dietary supplements in animal diets, and as consumer deodorizers in pet litters^10^,^11^,^12^,^13^,^14^,^15^,^16^,^17^. Very important application of ZLs is their use as hydroponic substrate for growing plants on space missions^18^, and as efficient light harvesting system in solar cells^19^.

ZLs are crystalline aluminosilicates consisting of oxygen-sharing SiO_4_ and AlO_4_ tetrahedral groups united by common vertices in three-dimensional framework and containing pores with diameters from 0.3 to 1.2 nm. The structural formula is M_x/m_ Al_x_Si_2-x_O_4_•nH_2_O, where m is the valence of cation M (K, Na, Ca, Mg), n is the water content, and 0 ≤ x ≤ 1. The porous space of ZL is occupied by exchangeable cations and water, and this is very important for ZL characterization since the localization, number and sizes of cations have an influence on the pore structure. Different research groups reported more than forty naturally occurring zeolites, and the clinoptilolite type zeolite (CZ) is one of the world’s most abundantly occurring and widely used zeolite minerals. CZ has unique physical-chemical properties, such as large amount of pore spaces, high dispersibility in both aqueous and organic solutions, high resistance to extreme temperatures and chemically neutral basic structure. CZ belongs to heulandite family of natural zeolites and has the following general chemical formula: (Na, K, Ca)_4_Al_6_Si_30_O_72_·24H_2_O^20^. The ratio between silicon and aluminium (Si*/*Al) of the CZ varies from 4.0 to 5.3. The presence of several types of porosity is an important feature of CZ. The microporosity (primary porosity) is caused by the specific crystal building of zeolite mineral grains. And the system of meso- and macropores (secondary porosity) is related to sizes and structural features of CZ grains in the rock^21^.

CZ is widely used in medicine and pharmacy as a biochemical sieve, feed and food additive, gas and odour absorber. It is inert, its purified form has demonstrated good stability in passage through the stomach, and the pharmacological and clinical studies have permitted to establish that this material does not produce biological damage to humans^22^,^23^,^24^. Based on numerous studies, CZ and their modified forms have been evaluated as a gastric antacid, anti-diarrheic, antihyperglicemyc, hipocholesterolemic and as a matrix for the loading and release of ions and organic molecules. Clinically formulated CZ in powder or liquid form is commercially available in the USA and Europe.

Generally, ZL behaviour and physical properties are studied by SEM, AFM, X-ray and optical spectroscopies. However, the mechanisms by which ZL materials have achieved success in pharmacology and health-care are not well understood, and optical imaging tools applied to ZL can help to understand some of mechanisms of these processes and make a significant contribution to biomedical and pharmacological applications of ZLs. Photoluminescence of natural ZLs were observed and investigated previously^25^,^26^,^27^,^28^, and it was shown that the majority of ZL types had characteristic excitation spectrum in 220–420 nm range with the maximum position around 300 nm, and emission spectrum in 280–600 nm spectral range. Furthermore, it was shown that luminescent properties of ZL were very much dependent on imperfections (or local defects) in crystals^25^. In most cases substitution of Fe^3+^ ions either singly or in association with other ions formed the centres responsible for luminescence, and the consistent correlation in the principal emission intensity around 360 nm with the iron concentration in the specimen was observed. This happened when Fe^3+^ ions substituted A1^3+^ ions in the tetrahedral aluminosilicate framework. In addition to this de-alumination, the following imperfections and factors also could be responsible for luminescence of ZL: phonon, electron and hole excitons, interstitial atoms and ions, decationization and dehydroxylation^25^. Moreover, it was shown that the photoluminescence intensity increased with the enrichment of clinoptilolite in row zeolites or when the CZ samples were irradiated by low dose of accelerated electrons with 8 MeV energy^27^. The appearance of new luminescence centres was explained by radiation-induced change of initial charge states of the lattice ions and formation of oxygen-cation vacancy. Contrariwise, ZL luminescence was effectively quenched by Na^+^ or Ca^2+^ ions located in the close vicinity to the luminescence centre^25^.

However, in the UV spectral region the linear absorption and scattering of bulk zeolites samples are very strong and non-favourable to perform conventional optical imaging or spectroscopic measurements. Here, the first effort of nonlinear optical imaging and quantitative study of loading and release of photodynamic dyes by micro- and nano-size CZ particles using multiphoton microscopy (MPM) and optical spectroscopy is presented.

## Results

### Multiphoton imaging of clinoptilolite-type zeolite

The sorption properties and pore size distribution of natural clinoptilolite have been investigated previously. It was demonstrated that for CZ, regardless of the mineral deposit, the value of specific surface area was in the range of 11–16 m^2^/g, the mineral is geometrically heterogeneous and possesses broad pore size (R) distribution with the characteristic two narrow (R < 2 nm and 2 < R < 5 nm) and one broad (R > 5 nm) domains^21^.

**Figure 1:**
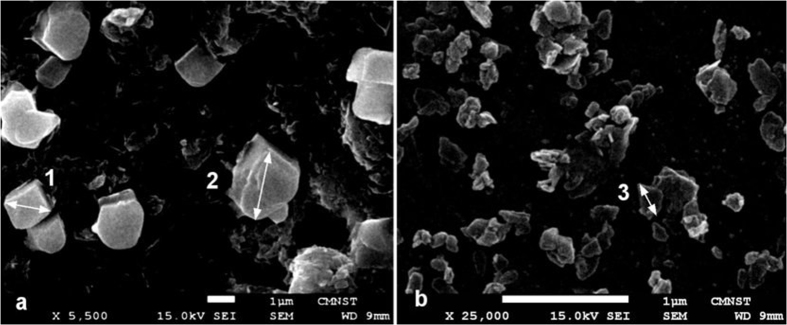
SEM images of morphology structure of micro-size (**a**) and nano-size (**b**) clinoptilolite. Scale bars are 1 μm.

To evaluate morphology and sizes of CZ particles, we performed SEM imaging of samples used in our experiments, and results for micro -and nano–size particles are presented in Fig. 1. Micro-size CZ particles have face shapes like a rectangle (e.g. particle 1, diagonal −1.71 μm), however, most of particles have curved morphology (e.g. particle 2, characteristic size −2.6 μm). CZ nano-particles have tablet-like shapes, and the characteristic length of typical particle is about 270 nm (see particle 3). Some aggregation of particles took place during sample preparation.

**Figure 2:**
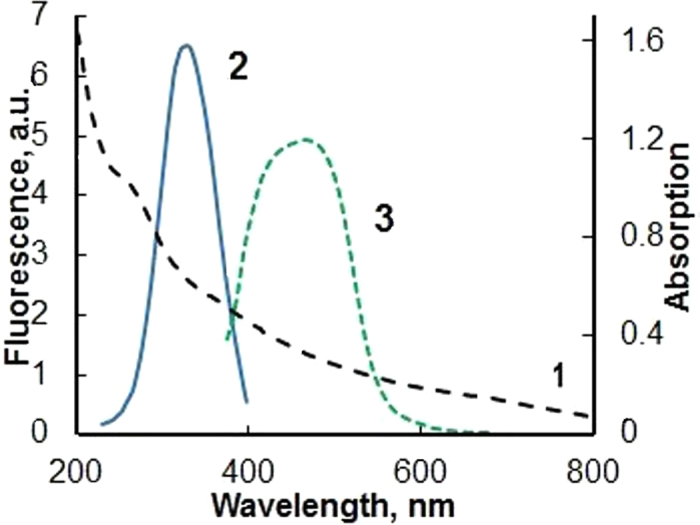
Absorption (1), luminescence excitation (2, λ_em_ = 420 nm) and emission (3, λ_exc_ = 360 nm) spectra of water suspension of CZ nanoparticles. CZ concentration is 1.25 mg/ml.

Linear absorption and luminescence spectra of nano-size CZ particles presented in Fig. 2.

**Figure 3:**
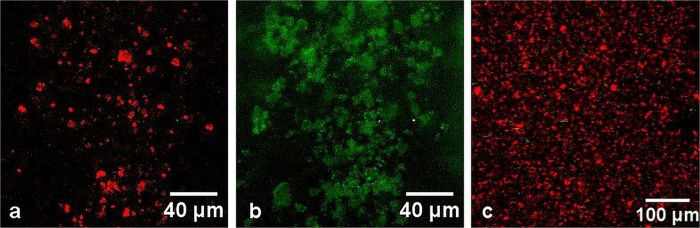
Multiphoton imaging of CZ micro particles on the surface of the microscope slide (**a**,**b**) and native mineral at the depth of 60 μm (**c**). Laser excitation wavelength: λ_exc_ = 780 nm (**a**,**c**), and λ_exc_ = 740 nm (**b**), Laser intensity: 10 mW. Green pseudocolour is used for TPEL (detection bandwidth 420–650), and red pseudocolour is used for SHG (detection bandwidth 380–400 nm). Objective: 40×/NA 1.2.

**Figure 4:**
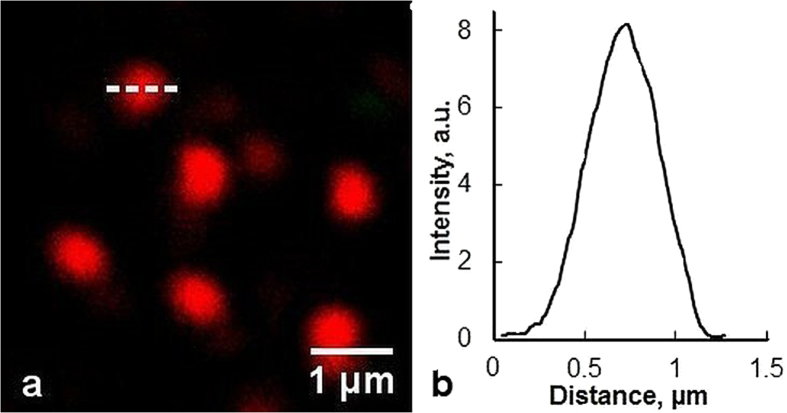
Multiphoton imaging of CZ nanoparticles on the surface of the microscope slide (**a**) and SHG intensity profile (**b**) of a single CZ nanoparticle along the dashed line. λ_exc_ = 780 nm, objective-40×/NA 1.2.

Further measurements showed that emission spectra of CZ intensified and moved to the short wavelength with the shortening of excitation wavelength (data not shown). Application of MPM for imaging of unstained natural CZ revealed that CZ micro- and nano-particles produced nonlinear optical response at near-infrared femtosecond laser excitation. At laser excitation with wavelength in 760–800 nm spectral region, a strong signal was registered in the 380–400 nm registration channel (see Fig. 3a). No signal was registered at excitation in 745–755 nm and 805–840 nm spectral regions, and comparatively weak signal was detected in the 420–650 nm channel when excitation wavelength was <745 nm (Fig. 3b). These images could be interpreted as the second harmonic generation (SHG) in CZ particles at 760–800 nm excitation (Fig. 3a) and formation of two-photon excited luminescence (TPEL), when excitation wavelength was shorter than 745 nm (Fig. 3b). Multiphoton imaging was also performed for native zeolite samples (Fig. 3c) and imaging depth reached up to 80 μm. The diameter of nano-size CZ particles were estimated to be <450 nm using SHG imaging (Fig. 4).

Observed nonlinear effects allowed to image individual zeolite particles and their interaction with dyes and biomolecules in different environments.

### Adsorption of dyes by CZ

Taking into account numerous biomedical applications of CZs, a series of experiments was performed to characterize CZ adsorption properties concerning to photodynamically active dyes, such as: hypericin (Hyp), methylene blue (MB), chlorin e_6_, Al-phthalocyanine and fluorescein. These dyes have been proved as anticancer-antimicrobial agents^29^,^30^. Therefore, for pharmaceutical application, it is important to study efficiency of CZ loading with these dyes and possibility of their release in different environmental conditions.

**Figure 5:**
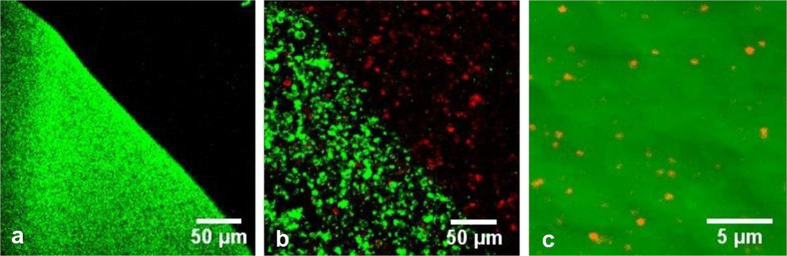
Multiphoton imaging of droplet of MB solution before (**a**) and after filling by CZ particles (**b**). (**c**) Multiphoton imaging of CZ particles in fluorescein solution. Green pseudocolour is TPEL (420–650 nm registration channel) and red pseudocolour is SHG (380–400 nm registration channel). Excitation wavelength λ_exc_ = 780 nm. Objective: 20×/NA 0.5.

It was shown that CZ particles adsorbed MB, chlorin e_6_ and Hyp molecules dissolved in PBS. In Fig. 5a green pseudocolour was used to visualize the part of droplet of MB solution on the microscope slide. After filling of the droplet by CZ particles (red spots in Fig. 5b indicated the SHG signal from individual CZ microparticles) MB molecules were adsorbed by CZ, and TPEL was seen from discreet particles. Simultaneously, the strong quenching of CZ SHG by MB molecules took place. Furthermore, Fig. 5c demonstrates that TPEL of fluorescein solution (green pseudocolour in Fig. 5c) has roughly uniform distribution after filling by CZ particles (red spots), and CZ does not adsorb and accumulate anionic fluorescein molecules in contrast to cationic MB molecules as shown in Fig. 5(b).

Similar multiphoton imaging showed that CZ effectively adsorbed photo-dynamically active Hyp and chlorin e_6_, but did not adsorb anionic Al-phthalocyanine molecules from aqueous solution. CZ structure has two parallel channels, *a* and *b*, which are connected to a third one, *c*. The size of the channels has dimensions of 3.1 × 7.5 Å, 3.6 × 4.6 Å and 2.8 × 4.7 Å correspondingly^20^. Due to the presence of the extra-framework, the dimensions of the dye molecules (>1 nm) are too large compared to enter of the channels. The outer surface of the CZ, which also includes the mesoporosity, and negative charge of CZ are the main parameters to be considered in the investigation of adsorption and desorption processes in CZ-dye systems.

### Kinetics of the Hyp release from CZ

**Figure 6:**
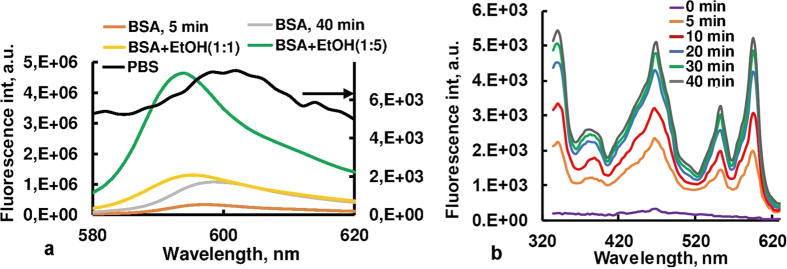
(**a**) Fluorescence spectra of the Hyp + CZ composite in PBS before (black line) and after treatment with bovine serum albumin (BSA) and ethanol (EtOH), λ_exc_ = 545 nm. Volume ratios of Hyp-BSA complex to ethanol are 1:1 (yellow line) and 1: 5 (green line). (**b**) Time-dependant recovery of excitation spectrum of Hyp in presence of BSA, λ_em_ = 650 nm.

Adsorption experiments using a batch equilibrium technique showed that suspension of CZ nanoparticles with concentration of 1.25 mg/ml were able to accumulate ~94% of Hyp molecules from aqueous solution of the dye with the concentration of 5·10^−6^ M. The Hyp + CZ composite was stable, and no release of Hyp was observed when the composite was diluted in PBS or distilled water. Hyp and Hyp + CZ composite in PBS had weak, non-structured, broadband fluorescence near 600 nm (see Fig. 6a, black line). However, the addition to the solution of ethanol (EtOH) or biomolecules such as bovine serum albumin (BSA), collagen, human haemoglobin and lipids, initiated a slow release of Hyp from CZ particles and increase in the Hyp fluorescence intensity. These results registered by fluorescence spectroscopy. The fluorescence spectra of Hyp + CZ system in PBS after addition of BSA (10^−6^ M) and different amounts of EtOH are presented in Fig. 6a, and an increase and spectral shift of the maximum in Hyp fluorescence spectrum are observed.

The release of Hyp and formation of Hyp-BSA complex with stronger fluorescence intensity occurred during the ~50-minutes after adding of BSA to the CZ-Hyp system in the phosphate buffered saline. Figure 6b demonstrates the recovery of Hyp fluorescence excitation spectrum after BSA addition to zeolite-Hyp system and desorption of Hyp from zeolite pores. The rate of Hyp release and interaction with BSA, as well as influence of EtOH on the Hyp fluorescence were investigated and quantified using kinetic spectrofluorometry, owing to the fact that Hyp fluorescence quantum yield dependent on molecular environment (see Fig. 6).

If the Hyp release from the CZ is assumed to be first order, then it follows the kinetic exponential decay model^31^ with the equation represented as:


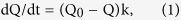


where Q is the amount of drug released in time t, Q_0_ is the maximum amount of drug released and k is the first order release constant. From equation (1):


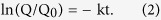


**Figure 7:**
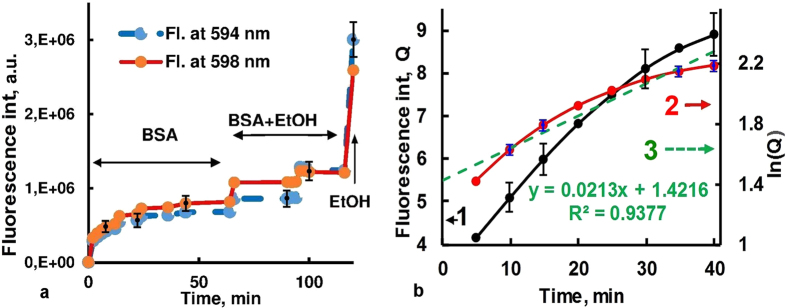
(**a**) Kinetics of Hyp florescence registered at 594 nm (maximum of fluorescence spectrum of Hyp-BSA complex) and 598 nm (maximum of fluorescence spectrum of Hyp in EtOH) at different concentration of EtOH, λ_exc_ = 545 nm. (**b**) The kinetic of BSA stimulated Hyp release (1), time dependence of logarithm of the Hyp fluorescence intensity (2), and the corresponding linear trend-line (3). The slope angle of line (3) gives the kinetic constant of Hyp release in the system.

Thus the plot of lnQ as ordinate and t as abscissa gives a straight line where the slope is the rate constant (k). The half-life T_1⁄2_ for the decay is given by T_1/2_ = ln2/k. The first order release rate constant, k, after fitting the experimental data with equation (2) is 0.021 min^−1^ for BSA (see Fig. 7b), and according to spectrofluorometric measurements, Hyp molecules released from CZ pores in the presence of BSA, collagen, human haemoglobin and lipids with the half-life T_1⁄2_ of 33.0, 30.1, 44.2 and 26.6 min, correspondingly.

## Discussion

Numerous examples of ZLs application in drug delivery and wound treatment have been reported in the literature^32^,^33^,^34^,^35^,^36^,^37^,^38^,^39^. Very important feature of zeolites for drug delivery is their mesoporosity^40^ that allows to combine diffusional pathways on different size scales and adsorb different molecules, nanoparticles and large biomolecules. Furthermore, modification of zeolites allows to change size of micropores and specific surface area without change of the zeolite skeleton^21^. These examples demonstrate that zeolites can be loaded or functionalized with drug molecules for different biomedical applications. Specifically, natural and sensitized ZLs have been used as hosts for small drug compounds, such as aspirin, doxorubicin, and paraquat molecules^32^,^33^,^34^,^35^,^36^. Furthermore, the loading, controlled delivery and release of anthelminthics and ibuprofen have been successfully demonstrated^37^. Clinoptilolite type of natural zeolite is safe for human. It is used in health industry as adsorbent of free radicals, toxic metals and radioactive elements due to exceptional adsorption, catalyst and ion-exchange properties. Moreover, it was reported a novel use of CZ as a potential adjuvant in anticancer treatment^38^,^39^.

Multiphoton microscopy allows to image natural zeolite samples in 3D and detect individual CZ nanoparticles in solution (Figs 3, 4 and 5). It opens new perspectives for investigating and understanding the interaction of host CZ particles with specific guest agents. In a general way, the results obtained in our study for the several photosensitizers indicate that the cause of the difference in the adsorptive behaviour is fundamentally related with the polarity of the molecules. Because of the excess of the negative charge on the surface of non-modified CZ, which results from isomorphic replacement of silicon by aluminium in the primary structural units, CZ effectively adsorbs cationic molecules and does not adsorb anionic molecules. The drug loading-release profiles is largely dependent on the cation content in the zeolite, and can be controlled by cation exchange.

More detailed research has been carried out on interaction of CZ with Hyp, a natural pigment found in plants of the Hypericum genus. Hyp has recently received increasing attention due to its high phototoxicity against viruses and anti-tumour photoreactivity^30^,^41^,^42^,^43^. In addition, it is shown that Hyp is an effective mediator for light-controlled selective modification of collagen in connective tissues, and may be used in biomedical engineering and therapy of collagen-related disorders^44^,^45^. However, Hyp in PBS and pure water tends to aggregate and form non-soluble and non-fluorescent complex thereby losing its tumour-selective properties^46^. Recently, the polyvinylpyrrolidone (molecular weights between 10 kD and 40 kD) has been suggested as a water soluble complex forming agent to prepare Hyp for photodynamic therapy and diagnostics^47^. For developing practical forms of applications of Hyp solutions for systemic use and delivery into body cavities, a new approach for Hyp delivery has been suggested here.

Addition of 20 μM BSA to CZ-Hyp system reduces the aggregation and precipitation, and changes Hyp absorption and fluorescence spectra. Under such conditions, ~140-fold higher fluorescence of Hyp in CZ-Hyp-BSA complex than in CZ-Hyp system is observed (see Fig. 6). Note that a mean pore diameter of non-treated original clinoptilolite (~3.9 nm) is smaller than all the principal dimensions of BSA, and adsorption of BSA to zeolite is minimal^48^. Moreover, NaCl and polyethylene glycol could be used to desorb the proteins adsorbed on zeolites without loss of activity^48^, and zeolite can be considered as biologically inert in these experiments. Furthermore, the Hyp emission spectrum after binding to BSA shows a maximum at 594 nm, whereas in water-ethanol environment the maximum observes at 598 nm (Fig. 6a). These spectral features of Hyp are in good agreement with literature data^49^,^50^ and allow to determine the local environment of Hyp molecules in multicomponent medium. Particularly, Figs 6 and 7 demonstrate a release of Hyp molecules from CZ and binding to BSA in the absence or at low concentration of EtOH and extrication from BSA at high concentration of EtOH. Thus, fluorescence measurements and optical imaging indicate that in aqueous solution CZ effectively adsorbs Hyp, and dye molecules are released from the CZ pores in the presence of biomolecules such as albumin, collagen, haemoglobin and lipids. The Hyp release rate is higher in the presence of EtOH, so it can be controlled by changing of concentration of EtOH or similar solvents. Such a behaviour is attributed to Hyp hydrophobic character that serves as driving force of release and redistribution of the dye.

## Conclusion

Our results indicate that CZ micro- and nano-particles render nonlinear optical response under the near-infrared femtosecond laser excitation. Dynamical interaction of CZ-dye complexes with the biomolecules is quantified using multiphoton microscopy, absorption and fluorescence spectroscopy, in that, it is proven CZ a potentially effective material for anticancer drug delivery and noninvasive optical control.

This research may open new perspectives in application of CZ in phototherapy and optical diagnostics, including noninvasive 3D multiphoton imaging, and help to introduce novel approaches into the clinical environment.

## Methods

The natural zeolite of the clinoptilolite type (CZ) originated from Noyemberyan, Republic of Armenia, was used in this study. Mineral identification using X-ray diffraction showed that the row zeolite consisted of ~87% clinoptilolite. The zeolite mineral was ground to 30–50 μm by using a mortar, then purified by washing with distilled water using a fluidized bed process, and dried at room temperature. Nano-sized CZ particles were obtained by grinding of minerals in a jet mill with subsequent sedimentation in aqueous solution. Particle size was then determined using a particle size analyser (Malvern Zetasizer 3000, UK). Most (98%) of the zeolite nanoparticles were smaller than 0.7 μm in diameter and the diameter of the 50% of zeolite particle (D50) was less than 0.3 μm. Photodynamic dyes (hypericin, methylene blue, chlorin e_6_, Al-phthalocyanine and fluorescein), biomolecules (bovine serum albumin, human haemoglobin, collagen, lipids) and chemical reagents were purchased from Sigma–Aldrich. All chemical reagents were of analytical grade (AR Grade) and used as received without further purification. Hyp powder was dissolved in dimethyl sulfoxide and stored at 4 °C. In experiments, the concentrated stock solution then diluted to 5 μM in phosphate buffered saline (PBS, pH 7.4). Other dyes were water-soluble and dissolved in PBS.

Optical properties of CZ, and their composites with dyes were characterized by “FluoroSENS” (Gilden Photonics Ltd. UK) spectrofluorometer and DU 800 UV/VIS (Beckman Coulter, Inc., US) spectrophotometer, with spectral resolution of 1 nm.

Multiphoton images were acquired by a system based on laser scanning microscope LSM 510 META (Carl Zeiss, Jena, Germany) coupled to the ti:sa fs Tsunami laser, operating at 730–850 nm wavelength interval, pulse-width of 120 fs, and repetition rate of 80 MHz. The *in situ* average power is between 5 to 15 mW. Detection bandwidths for second harmonic generation and two-photon excited luminescence signals are 380–400 nm and 420–650 nm, respectively. Plan Neofluar air objectives 20×/0.5 NA, working distance WD = 1.3 mm and Fluar water immersion 40×/1.2 NA, WD = 0.28 mm (Carl Zeiss, Germany) objectives are used in the multiphoton imaging.

Adsorption experiments were carried out using a batch equilibrium technique by placing adsorbent in a glass bottle containing of a dye solution at various concentrations. PBS was used as a solvent. The equilibrium concentrations of dyes were determined at 470 and 550 nm using the DU800 spectrophotometer. The calibration curves for each dye at the respective wavelengths were established as a function of dye concentration. After experiments, the adsorbent was separated from the solution by gravity, and the concentration of the remaining dye solution was obtained using calibration curves. The amounts of dye adsorbed were determined by the difference between the initial and remaining concentrations of dye solution. The adsorption capacity was calculated considering the concentration of adsorbed dye (mg/L), solution volume (L), and adsorbent mass (g). Distilled and deionized water with a conductivity value of 6 × 10^−8^ Siemen/cm was used in the experiments.

## Additional Information

**How to cite this article:** Hovhannisyan, V. *et al*. Photodynamic dye adsorption and release performance of natural zeolite. *Sci. Rep.* **7**, 45503; doi: 10.1038/srep45503 (2017).

**Publisher's note:** Springer Nature remains neutral with regard to jurisdictional claims in published maps and institutional affiliations.
